# Development and validation of a case definition for problematic menopause in primary care electronic medical records

**DOI:** 10.1186/s12911-023-02298-x

**Published:** 2023-10-05

**Authors:** Anh N.Q. Pham, Michael Cummings, Nese Yuksel, Beate Sydora, Tyler Williamson, Stephanie Garies, Russell Pilling, Cliff Lindeman, Sue Ross

**Affiliations:** 1 Department of Rehabilitation Medicine, University of Alberta, Edmonton, Canada; 2 Department of Community Health Sciences, University of Calgary, Calgary, Canada; 3 Faculty of Pharmacy and Pharmaceutical Sciences, College of Health Sciences, University of Alberta, Edmonton, Canada; 4 Department of Obstetrics and Gynecology, University of Alberta, Edmonton, Canada; 5 Department of Family Medicine, University of Calgary, Calgary, Canada; 6 Department of Family Medicine, University of Alberta, Edmonton, Canada

**Keywords:** Primary care data, Case definition, Electronic medical record phenotyping, Problematic menopause.

## Abstract

**Background:**

Menopause is a normal transition in a woman’s life. For some women, it is a stage without significant difficulties; for others, menopause symptoms can severely affect their quality of life. This study developed and validated a case definition for problematic menopause using Canadian primary care electronic medical records, which is an essential step in examining the condition and improving quality of care.

**Methods:**

We used data from the Canadian Primary Care Sentinel Surveillance Network including billing and diagnostic codes, diagnostic free-text, problem list entries, medications, and referrals. These data formed the basis of an expert-reviewed reference standard data set and contained the features that were used to train a machine learning model based on classification and regression trees. An ad hoc feature importance measure coupled with recursive feature elimination and clustering were applied to reduce our initial 86,000 element feature set to a few tens of the most relevant features in the data, while class balancing was accomplished with random under- and over-sampling. The final case definition was generated from the tree-based machine learning model output combined with a feature importance algorithm. Two independent samples were used: one for training / testing the machine learning algorithm and the other for case definition validation.

**Results:**

We randomly selected 2,776 women aged 45–60 for this analysis and created a case definition, consisting of two occurrences within 24 months of International Classification of Diseases, Ninth Revision, Clinical Modification code 627 (or any sub-codes) OR one occurrence of Anatomical Therapeutic Chemical classification code G03CA (or any sub-codes) within the patient chart, that was highly effective at detecting problematic menopause cases. This definition produced a sensitivity of 81.5% (95% CI: 76.3-85.9%), specificity of 93.5% (91.9-94.8%), positive predictive value of 73.8% (68.3-78.6%), and negative predictive value of 95.7% (94.4-96.8%).

**Conclusion:**

Our case definition for problematic menopause demonstrated high validity metrics and so is expected to be useful for epidemiological study and surveillance. This case definition will enable future studies exploring the management of menopause in primary care settings.

## Background

The menopause transition is a normal stage in a woman’s life, generally occurring around the age of 51 in North America (with a typical range between 45 and 55 years), when the production of hormones (i.e., estrogen, progesterone) from the ovaries gradually decline and women cease menstruation [1]. Menopause can also occur as a result of surgery that removes both ovaries, for example, for women who are at risk of ovarian or breast cancer [2]. Most women experience menopause without significant difficulty. For others, menopause symptoms can be severe and debilitating and greatly affect quality of life [3]. Among women referred to a menopause clinic in a large urban centre in Alberta, Canada, self-reported moderate or severe menopause symptoms included sleep disorders (76%), night sweats (51%), hot flashes (50%), mood swings (48%), and depression (38%) [4]. Symptom severity is associated with increased risk of long-lasting physical illness such as type-2 diabetes, osteoporosis, and cardiovascular diseases. In addition, the menopause transition, and especially during perimenopause, is associated with increased risk of developing depressive episodes, particularly among women who previously suffered from depression [5]. Female suicide rates in western countries peak around the age of menopause [6], which is considered a vulnerable period for women with a history of depression [5]. Insufficient treatment of symptoms can lead to worsening symptoms making proper diagnosis and treatment a priority for menopausal women [3].

Most women experiencing difficult menopausal symptoms will first consult their family physicians for help, but little is known about the epidemiology or clinical management of this condition in a primary care setting. Symptomatic women usually present with more than one menopause symptom [7] making a clear definition for problematic menopause challenging.

A literature search to determine how problematic menopause is defined found only three papers [8–10], and none were focused on primary care. Therefore, additional research is needed to understand the actual burden of menopausal symptoms in primary care and to consider whether service provision could be improved.

The Canadian Primary Care Sentinel Surveillance Network (CPCSSN) database contains relevant clinical data extracted from electronic medical records (EMRs) held by primary care practices across Canada. Because physicians record these data for clinical and administrative purposes, and not for research, there is a need to develop and validate case definitions (i.e., rules for identifying patients with a condition). CPCSSN has reported on definitions for many chronic conditions [11–16], but not for problematic menopause. The aim of this study was to use CPCSSN data to develop and validate a case definition of ‘problematic menopause’ in women who visit their family physicians.

## Methods

### Data source

CPCSSN is a collaboration of practice-based, primary care surveillance and research networks across Canada that work to better understand the epidemiology and management of chronic health conditions to improve patient care [17], [18]. It collects and combines deidentified, patient level, primary care EMR data from participating practices across the country. CPCSSN extracts a variety of data, including: billing and diagnostic codes, diagnostic free-text, medical history and problem lists, medication prescriptions, lab results, physical exam values (e.g., BMI, blood pressure), risk factors (e.g., smoking status), vaccines administered, and referral specialties (see [19] for a more comprehensive listing). As of 2023, the CPCSSN database does not include physician notes. Raw EMR data are cleaned, standardized, and deidentified to create a research-ready data set that is used for multiple purposes, including population health research and surveillance. CPCSSN data can go back as far as the 1990s, although the data are more complete starting in the late 2000s, as this is when EMR use became more widespread in Canada. In general, patients with data recorded in CPCSSN are slightly older and more likely to be women than the Canadian general population [20].

To date, CPCSSN case definitions have been developed and validated for 28 case definitions including: chronic obstructive pulmonary disease (COPD), dementia, depression, diabetes mellitus, type-1 diabetes, diabetic nephropathy, epilepsy, hypertension, osteoarthritis, parkinsonism, speech disorders, herpes zoster, pelvic floor disorders, adult asthma, pediatric asthma, among others with validation metrics ranging from good to excellent [11–16].

We used data from multiple tables within the CPCSSN data set, including: the Billing table which contains claims data submitted to the province, the EncounterDiagnosis table which contains diagnoses recorded during a visit, the HealthCondition table which contains patient medical history and problem list data, the Medication table which contains medications prescribed by family physicians, and the Referral table which contains the services to which patients are referred (e.g., gynecology).

### Study sample

In the CPCSSN database extracted up to December 31, 2020, there were 211,103 women aged 45–60; however, only 190,392 (90%) had at least one clinical record in the specific CPCSSN data tables used in this study. A preliminary search for problematic menopause in the dataset using a combination of an International Classification of Diseases, Ninth Revision, Clinical Modification (ICD-9-CM) code for menopause (627*) and menopausal hormone therapy (i.e., estrogen and progesterone) yielded an estimated prevalence of 19%. The sample size, *N*, for the validation set was determined using the Wald 95% confidence interval (CI) formula [21]:

1 $$N={1.96}^{2}\frac{{S}_{n}\left(1-{S}_{n}\right)}{{\left(\frac{c}{2}\right)}^{2}p}$$

where *S*_*n*_ is the expected sensitivity of the case definition, *c* is the full width of the CI for the sensitivity, and *p* is the expected disease prevalence within the sample. To provide validation metrics of at least 80% sensitivity and a 95% CI of no more than 10% full width, a sample of 1,388 patients was required for the validation set. We selected the same number of patients for the training data set, as this was expected to provide at least 250 positive cases of problematic menopause to test and train the machine learning (ML) algorithm. Thus, the total sample consisted of 2,776 randomly selected patients. The data were selected to be representative of the CPCSSN data holdings across sex, regions, and EMRs.

### Reference data set

We used previous CPCSSN methods for other chronic conditions to conduct the development and initial validation of a case definition for problematic menopause [22–24]. A working set of criteria, developed by our team and a clinical expert in menopause research and mature women’s health (NY), was used as guidance to support chart reviewers in labeling charts. It incorporated four types of information: ICD-9-CM diagnostic codes, which are used in Canada when physicians submit billings for their medical services to provincial / territorial governments and to record diagnoses within patient charts, textual descriptors of menopausal disorders, relevant drugs, and relevant referrals. The working set of criteria incorporated the following elements:

**ICD-9-CM** diagnostic code 627 and relevant sub-codes for menopausal and postmenopausal disorders.

**Textual descriptors** of menopausal symptoms were identified in collaboration with a clinical expert in menopause research (NY). The text descriptors included the terms ‘menopaus*’ and ‘climacteric’, where the asterisk indicates any character (e.g., menopause, menopausal). Abbreviations, alternative spellings, synonyms and other relevant variations of these terms were also included.

**Relevant drugs** used for problematic menopause were identified using e-CPS, the online Compendium of Pharmaceuticals and Specialities [25]; e-CPS was used to confirm the specific indications and availability of drugs in Canada for the management of menopause. The drug descriptions included current and recently discontinued generic and proprietary names and were coded to Anatomical Therapeutic Chemical (ATC) classification codes.

**Referral** for problematic menopause to a specialist menopause clinic or gynecology.

Using the working set of criteria, five trained clinical chart reviewers inspected the CPCSSN records in the patient sample to create a reference standard data set. The aim was to judge if the physician had already made a diagnosis, by looking into all available clinical information, including diagnosis codes, short (under 255 characters) diagnostic free-text, prescribed medications, and referrals. It was set clear to reviewers that one single diagnosis code or prescription alone was not sufficient to make a diagnosis, additional evidence was required. If a chart reviewer was uncertain of a patient’s classification, the opinion of a clinical expert (NY) was sought for adjudication. Fleiss’s Kappa, which is a statistical score used to assess consistency between multiple reviewers (i.e., inter-rater reliability) [26], was calculated on a subset of the reviewed charts.

### Machine learning algorithm

**Fig. 1:**
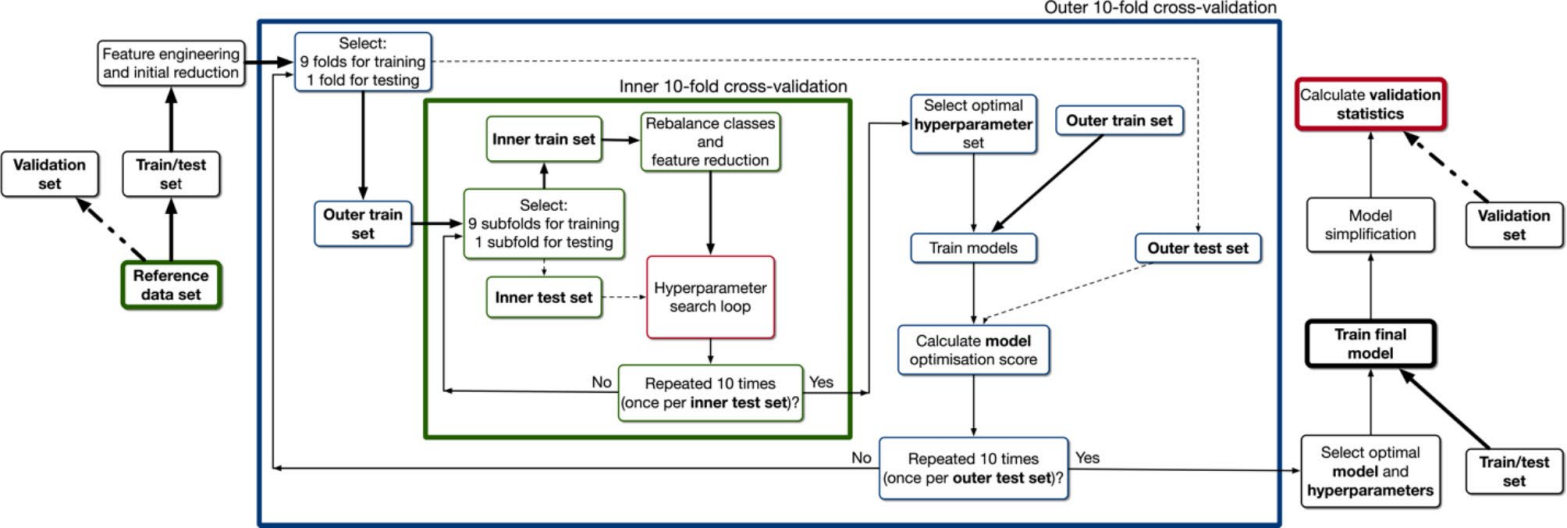
ML algorithm used for case definition creation. The larger (blue) and smaller (green) squares contain the processes that occur during nested 10-fold cross-validation

Case definition creation and assessment was accomplished via the algorithm outlined in Fig. 1. Once the reference data set was finalised, it was divided into two random samples: a train / test set (*n* = 1,388) that served as the labelled data source for a machine learning algorithm, and a validation set (*n* = 1,387) that allowed independent assessment of the accuracy of the final model. The train / test set was then input into a feature engineering algorithm which selected those elements (i.e., variables or features) of the chart that were predictive of problematic menopause. We extracted all possible features from multiple fields in each patient’s CPCSSN data that included billing and encounter diagnosis ICD-9-CM diagnosis codes and free-text, patient problem list ICD-9-CM diagnosis codes and free-text, referral free-text, and prescribed medication ATC codes. Our expert advisors believed lab results and physical exam values would be much less informative than the other included features, and so these data were excluded. Free-text was extracted using a “bag-of-words” approach, one feature being generated for each possible word or each possible pair of words (bi-grams) across all records. Fifty different negated or uncertain term constructions (e.g., ‘not menopause’, ‘menopause?’, ‘rule out menopause’) were used to avoid including false or ambiguous diagnoses. To reduce the total number of features generated, only those existing in the CPCSSN record of at least ten patients were selected. Unique features were created for one or more instances of each word or code, two or more instances within 12 consecutive months, two or more instances within 24 consecutive months, and two or more instances within the entire patient chart. All features were treated as binary variables (i.e., the patient has or does not have the feature).

This process generated over 86,000 unique features. Since only a small number of these were expected to be predictive of menopause, we used a simple information measure to assign an importance to each feature: the difference between the number of true positives (have the feature and condition) and the number of false positives (have the feature, but not the condition) over the total number of patients who have the condition. The 300 features with the highest values were selected for input into the supervised ML algorithm.

A supervised ML approach was applied to determine what data elements (i.e., features) among the charts in the sample were most relevant to creating a case definition for problematic menopause. We selected the Classification and Regression Trees (CaRT) algorithm, as implemented by the python scikit-learn package, for this purpose [25, 26], as it outputs a clear, human-readable, rules-based classification [27], as opposed to black-box algorithms like those based on random forests, boosted trees, support-vector machines, or neural networks. The CaRT algorithm uses features within the data as rules for how to classify patients into binary groups (i.e., patients with or without a given set of features). The result is a ‘tree’ of rules - a decision tree - indicating how to identify patients with a given outcome (e.g., problematic menopause). The algorithm has several tunable hyperparameters that determine how deep and wide the final decision tree becomes [27]. Of these, we allowed the branching criterion, branching strategy, maximum tree depth, minimum samples required per branch, and class weights hyperparameters to be varied during training.

Training of the machine learning model was completed using nested 10-fold cross-validation (NCV), as it has low inherent bias and enables simultaneous optimisation of ML algorithm hyperparameters and training of the classification model [28]. This process is pictured inside the large blue box in Fig. 1. In the outer loop, training of the ML model occurs. The data are partitioned into ten samples, or folds, of the same size; at each iteration of the process, nine folds are used to train the ML model and one to test it (i.e., calculate some measure of accuracy). In the inner loop, hyperparameter optimisation occurs. The nine-fold training set from the outer loop is divided into ten more equal sized folds; again, nine are used to train the ML algorithm and one is used to calculate an optimisation metric that is used to determine the best hyperparameter set. The inner loop is repeated ten times total, and a hyperparameter set is selected as one with the highest average optimisation metric over all ten iterations. This hyperparameter set is then used by the outer loop to train the ML model. This process is repeated nine more times, using a different test sample at each iteration.

We selected the F_1_-score [29] as the optimisation metric for both the inner (hyperparameter optimisation) and outer (model training) cross-validation loop as it incorporates sensitivity, *S*_*n*_, and positive predictive value, PPV, in a symmetric way.

2 $${F_1} = 2{\left( {\frac{1}{{{S_n}}} + \frac{1}{{PPV}}} \right)^{ - 1}} \cdot$$

Thus, this score gives equal weight to false positives, through the PPV, and false negatives, via the sensitivity. By maximising the F_1_-score, the algorithm attempts to jointly maximise sensitivity and PPV by effectively minimising false positives and false negatives. Since true negatives are not part of the F_1_-score, specificity will not be optimised, and so sensitivity and PPV may be increased at the expense of reduced specificity. We have selected this approach so that the resultant case definition will be suited to epidemiological research (high sensitivity) and, potentially, positive case identification (high PPV).

Training and testing data were split using stratified sampling, so that the ratio of cases to non-cases was equal in each fold. To reduce possible bias due to unequal class-sizes (i.e., case-to-non-case ratios different from unity) [19], at each iteration of the NCV algorithm, we trained the model using four different sampling approaches: random under-sampling, random over-sampling, combined random under- and over-sampling, and unchanged (no sampling). The first three methods artificially increase the prevalence of problematic menopause cases within the sample by randomly duplicating existing cases (over-sampling), randomly removing non-cases (under-sampling), or through a combination of the two. We also employed two different feature reduction algorithms, recursive feature elimination (RFE) [30] and a clustering algorithm known as *k*-best feature selection (kBF) [31], to further reduce the feature set from 300 down to a few tens of the most important features. This greatly reduces algorithm runtime with little impact on case definition accuracy, as most features are essentially uninformative. Models were trained for each sampling method / feature reduction algorithm pair and for two different orders of operation: feature reduction first and sampling second, and vice versa.

The optimal hyperparameters and sampling method / feature reduction algorithm pair were selected as the set that produced the highest average validation metrics (sensitivity, specificity, PPV, negative predictive value (NPV)) across all folds of the outer 10-fold cross-validation. These parameters were then used to train the CaRT algorithm on the entire train / test data set (all *n* = 1,388 charts) and to create a provisional case definition. We found that the case definition generated by the CaRT algorithm for problematic menopause was quite complex because of the number of rules required. To determine if it could be simplified, we selected the most relevant features from the provisional case definition by ranking them by their importance scores, a measure which indicates the relative importance of each individual feature to the accuracy of the model [32]. We then created case definitions using each individual feature and out of each pair of features. The final case definition chosen was the one with the highest validation metrics (sensitivity, specificity, PPV, NPV) as determined from the entire train / test data set. An independent validation data set (*n* = 1,387) was then used to calculate the case definition validation metrics, effectively assessing the generalisability of the case definitions to new data. All steps of this analysis were coded in Python 3.7.

## Results

From 190,392 women aged 45–60 as of December 31, 2020, with at least one clinical record in the CPCSSN tables of interest, we selected a random subset of 2,776 EMR records for chart review. Table 1 presents the demographic characteristics of these patients. For the measures presented, the cohort is broadly similar to the CPCSSN population in both age distribution, rurality, and burden of disease.

**Table 1:** Demographic and clinical characteristics of the patient sample

Baseline characteristics	Chart review cohort (N = 2,776)	CPCSSN 2020 dataset (N = 190,392)
**Age**, n (%)		
45–49 years	802 (28.9)	58,636 (30.8)
50–54 years	806 (29.0)	57,630 (30.3)
55–60 years	1168 (42.1)	74,126 (38.9)
**Urban residence**, n (%)	2,341 (84.4)	150,915 (79.3)
Number of chronic conditions*, n (%)		
0	966 (34.7)	72,509 (38.0)
1	765 (27.6)	43,743 (23.0)
2	421 (15.2)	28,097 (14.8)
3+	624 (22.5)	46,043 (24.2)

* Based on 11 of the conditions that CPCSSN has developed and validated definitions for: chronic kidney disease, COPD, dementia, depression, diabetes mellitus, dyslipidemia, epilepsy, Herpes Zoster, hypertension, osteoarthritis, Parkinson’s disease [11–14]

**Fig. 2:**
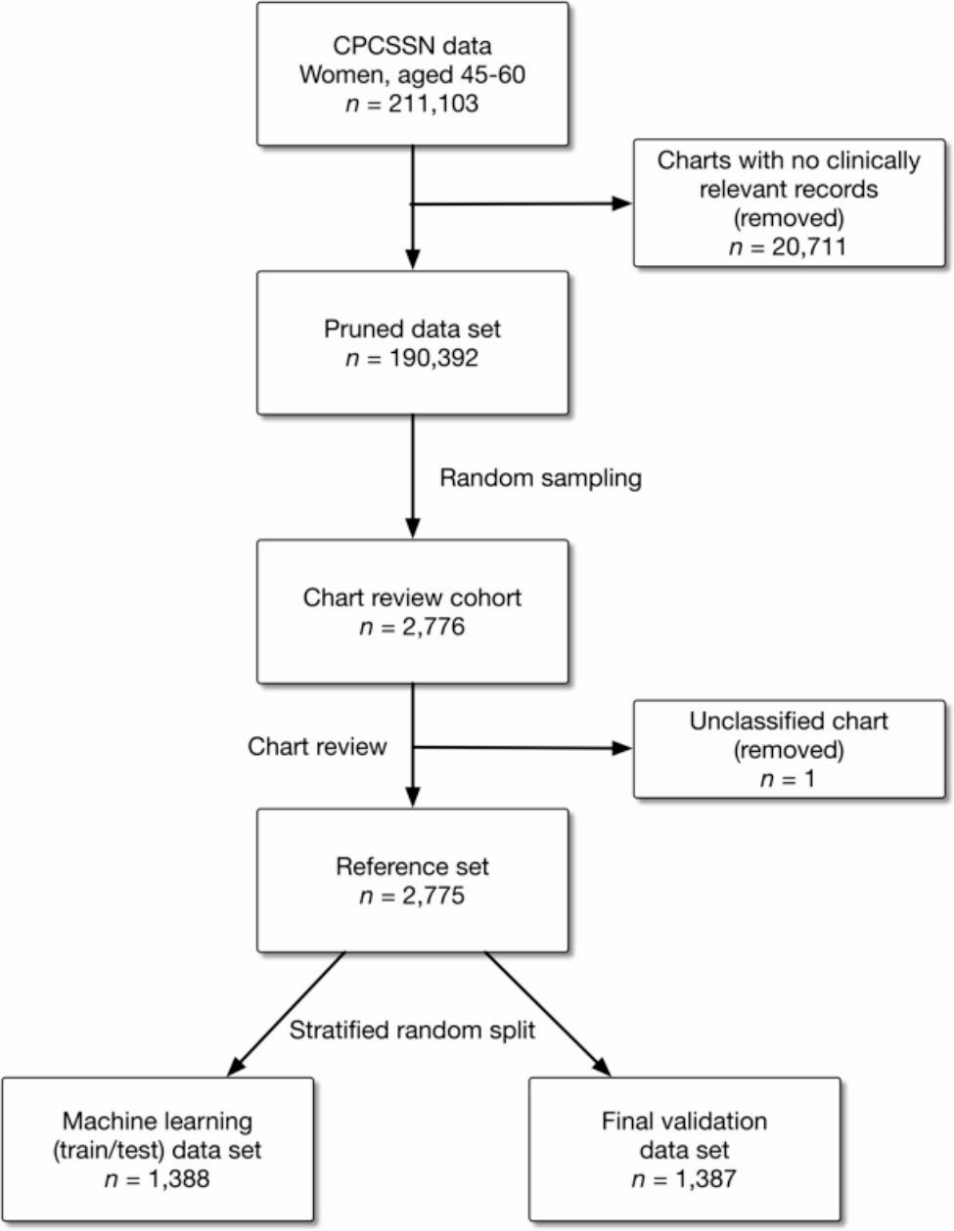
Flowchart for the patient chart selection process

Five reviewers were tasked with inspecting the EMR charts of 2,776 patients to create the reference standard data set. Charts that reviewers were unable to classify were independently inspected by two additional reviewers (SR and AP). The independent reviewers did not agree on ten charts and those were sent to a certified menopause practitioner (NY) for a final review. One record did not include sufficient information to be classified and was excluded from the analysis. Figure 2 presents the steps and number of charts selected during this process.

The chart review process identified 533 out of 2,775 women (19.2%) in the reference set as having problematic menopause. We also assessed the reviewer inter-rater reliability using Fleiss’s Kappa, *κ*, on a subset of 120 charts and found *κ* = 0.84, indicating that the reviewers were in good to excellent agreement.

We applied the nested cross-validation (NCV) algorithm to the train / test data set for 14 different combinations of feature reduction / resampling method pairs and the resultant 10-fold averaged optimisation metrics are shown in Table 2. We found that applying different weights for each outcome class (i.e., cases and non-cases) using the built-in class weights hyperparameter of the CaRT algorithm improved the performance of our models much more than by applying standard resampling techniques. The performance of the ML algorithms was typically reduced when resampling methods (i.e., under- or over-sampling) were applied to the data and the two best performing models did not employ any resampling. For the same sampling method, the recursive feature elimination (RFE) technique tended to produce higher (better) F_1_-scores than for the *k*-best feature (kBF*)* selection algorithm. Comparing the F_1_-scores when applying resampling first and feature reduction second to feature reduction first and resampling second, the RFE method produced higher metrics if feature reduction was completed before resampling, while the results for kBF are more mixed. Overall, the optimal performance was achieved by applying RFE with no resampling (bold row in Table 2). The order of the methods in the first table cell indicates the order in which the methods were applied in the algorithm.

**Table 2:** Optimisation metrics for models created by resampling training data and applying feature reduction. Abbreviations: RFE = recursive feature elimination, kBF = *k*-best features

Resampling & Feature Reduction Method		Sensitivity % (95% CI)	Specificity % (95% CI)	PPV % (95% CI)	NPV % (95% CI)	F_1_-Score
random over- & under-sampling;	RFE	90.3 (78.7–99.1)	85.7 (77.5–93.4)	59.8 (49.5–74.8)	97.6 (94.7–99.8)	0.720
random over-sampling;	RFE	87.5 (74.8–96.0)	85.9 (79.6–94.3)	59.9 (50.2–76.6)	96.9 (93.9–98.9)	0.711
random under-sampling;	RFE	93.0 (85.4–96.2)	83.5 (80.1–89.8)	56.3 (51.1–66.2)	98.2 (96.4–99.0)	0.701
RFE;	random over- & under-sampling	80.1 (66.2–88.4)	92.0 (85.6–96.1)	70.1 (57.0-82.9)	95.4 (92.2–97.2)	0.748
RFE;	random over-sampling	79.3 (66.2–88.4)	93.5 (88.2–98.5)	74.0 (60.1–93.0)	95.2 (92.2–97.4)	0.766
RFE;	random under-sampling	79.3 (66.2–88.4)	92.5 (88.0-96.1)	70.9 (60.1–82.9)	95.2 (92.2–97.2)	0.749
**RFE;**	**None**	**81.2** **(70.1–91.3)**	**95.0** **(92.9–97.9)**	**78.6** **(69.5–89.9)**	**95.7** **(93.1–98.0)**	**0.799**
random over- & under-sampling;	kBF	86.0 (73.1–95.2)	84.9 (77.8–91.7)	57.1 (47.0-69.2)	96.5 (93.3–98.7)	0.686
random over-sampling;	kBF	87.6 (73.1–99.1)	85.1 (76.3–93.7)	58.4 (47.2–76.1)	96.9 (93.4–99.8)	0.701
random under-sampling;	kBF	87.9 (77.8–99.1)	86.6 (77.3–94.1)	60.9 (49.5–76.2)	97.0 (94.5–99.8)	0.720
kBF;	random over- & under-sampling	80.4 (59.8–96.1)	88.2 (80.6–97.0)	63.1 (52.4–81.6)	95.4 (91.6–98.9)	0.707
kBF;	random over-sampling	81.6 (59.8–99.1)	87.8 (78.4–96.4)	62.4 (49.9–80.1)	95.7 (91.6–99.8)	0.707
kBF;	random under-sampling	84.4 (67.8–99.1)	87.3 (79.1–98.1)	62.7 (50.7–91.3)	96.3 (92.5–99.8)	0.719
kBF;	None	61.2 (39.3–79.9)	97.4 (92.5–99.1)	85.4 (69.3–94.0)	91.9 (87.5–95.4)	0.713

A provisional case definition was generated by training the CaRT algorithm on the entire train / test data set, with feature reduction completed using RFE and without any resampling. The rules for the case definition were rather complex, with 67 different rule sets required to classify whether or not each patient had problematic menopause. To simplify this rule set, we calculated the importance scores of all nine features that make up the provision case definition. These are shown in Table 3. We then created nine case definitions consisting of each individual feature in the provisional definition and 45 more case definitions from each pair of features. The final case definition was selected as the one generating the highest validation metrics when applied to the entire train / test data set.

**Table 3:** Top nine most important features in the CaRT case definition

Feature	Code Description	Data type	Importance score
One ATC code G03CA (or any sub-codes)	Natural and semisynthetic estrogens	Medication	0.54534
Two ICD-9 codes 627 (or any sub-codes) in 24 months	Menopausal and postmenopausal disorders	Billing, encounter diagnosis or problem list	0.23344
Two ATC codes G03 (or any sub-codes)	Sex hormones and modulators of the genital system	Medication	0.07973
Two ATC codes G03CA (or any sub-codes)	Natural and semisynthetic estrogens	Medication	0.04548
Two instances of “menopausal” in free text		Billing, encounter diagnosis or problem list	0.03261
One code ATC code G03DA04	Progesterone	Medication	0.02862
Two ATC codes G03DA (or any sub-codes)	Pregnen (4) derivatives	Medication	0.01343
One instance of “postmenopausal” in free text		Billing, encounter diagnosis or problem list	0.01245
One ICD-9 code 627 (or any sub-codes)	Menopausal and postmenopausal disorders	Encounter diagnosis	0.00890

*ATC: Anatomical Therapeutic Chemical (ATC) Classification;*

*ICD-9: International Classification of Diseases, Ninth Revision;*

*“Or any sub-codes” means all codes starting with the same characters (e.g., G03 (or any sub-codes) includes G03A, G03B, G03CA, G03DA04, etc.)*

The final case definition consists of women aged 45 to 60 with a chart that contains at least one of the following two elements:

- two instances of ICD-9 code 627 (menopausal and postmenopausal disorders) or any of its sub-codes within 24 months in any of the following portions of the patient record.
  - billing, or.
  - encounter diagnosis, or.
  - problem list, or.
- at least one prescription for a medication in the ATC class G03CA (natural and semisynthetic estrogens).

As compared to the complex case definition generated by the CaRT algorithm, the final definition is both simple and clear. Both definitions were applied to the validation data set to provide an independent measure of their accuracy and generalisability (see Table 4). The final case definition has higher sensitivity and PPV than the CaRT-based definition, while the other validation metrics are quite similar.

**Table 4:** Validation metrics for the final case definition following application to the validation data set

	Sensitivity % (95% CI)	Specificity % (95% CI)	PPV % (95% CI)	NPV % (95% CI)
CaRT	79.2 (73.8–83.8)	92.7 (91.0-94.1)	70.9 (65.3–75.9)	95.2 (93.8–96.3)
Simplified case definition	81.5 (76.3–85.9)	93.5 (91.9–94.8)	73.8 (68.3–78.6)	95.7 (94.4–96.8)

## Discussion

The value of this research lies in producing a case definition that can provide the foundation for quality improvement initiatives in the primary care treatment of menopause in Canadian women and better knowledge about the burden of menopausal symptoms in the community. We believe this is the first such study in Canada, and the first to apply population health analytic methods to explore a case definition for problematic or treated menopause.

We found that resampling to correct for the class imbalance (more non-cases than cases) in the data set led to case definitions with lower validation metrics than those without re-balancing. This might indicate that an approximately four-to-one class imbalance ratio is not so severe as to meaningful effect the CaRT algorithm’s ability to discern between informative and uninformative features in the data set. Our findings also align with evidence suggesting that decision trees do not suffer from the same degree of bias from imbalanced data sets as other methods [27].

The provisional problematic menopause case definition, created through application of the CaRT algorithm, produced good validation metrics, with sensitivity, specificity, PPV, and NPV greater than 70%, more than sufficient accuracy for epidemiological purposes [11]. However, the decision tree was complex, requiring 67 different rule sets to classify patients, making the case definition challenging to interpret. Therefore, we extracted the small number of individual features that make up the case definition, nine in total, and created a simplified definition. This simplified definition also has good validation metrics, and is clinically and epidemiologically relevant, with slightly higher sensitivity (81.5% vs. 79.2%) and slightly improved PPV (73.8% vs. 70.9%) relative to the CaRT-based case definition.

Few studies have focused on the distribution of menopausal symptoms in the community or management of menopausal symptoms in primary care settings. Among published studies, most tend to concentrate on particular aspects of menopause management, such as vasomotor symptoms and menopausal hormone therapy (e.g., Kiran, 2022 [25]) rather than describing primary care management more generally. The development of this case definition for problematic menopause offers future opportunities to better understand the primary care management of menopause. For example, it can be used to estimate and predict the workload associated with providing care for patients who have problematic menopause by family physicians and other primary care team members. As a result, primary care teams may be able to streamline their work, perhaps providing additional resources for menopausal women, such as introducing multidisciplinary menopause clinics that might off-load physician workload [4]. Using this CPCSSN case definition, our next study will explore whether the treatment of Canadian women is optimal as recommended by the Society of Obstetricians and Gynaecologists of Canada and/or North American Menopause Society guidelines for the management of menopausal symptoms [33]. Then we shall develop educational materials for distribution by the CPCSSN organisation to the individual primary care practice-based networks and enter into discussion with medical schools, professional organizations, and the College of Family Physicians of Canada to enhance physician education about menopause management.

### Limitation

A high-quality reference data set is vital for developing an accurate and useful case definition. While the extensive reviewer training and high Fleiss’s Kappa point to a high-quality reference set, the review process is never perfect, and so some charts will be mislabelled. Patient records used in this study consisted of billing data, diagnoses, problem lists, medications, and referrals - clinical notes, a rich source of diagnosis data, and complete surgical histories were unavailable. These additional data may have allowed identification of problematic menopause patients to be more accurate; hence, enabled creation of a more robust reference data set. This study reflects the medical data as recorded in family medicine practices and was used to develop case definition algorithms for problematic menopause. Our data does not include women who have not accessed family physician care: this ensures that women who are identified as “cases” will be those with menopausal problems sufficiently troublesome to seek family medicine health care, a more rigorous test of prevalence than self-report in a population survey. However, it may be that these patients do not represent all Canadian women; the more than 20% of Canadians who do not have a family physician or nurse practitioner [34], women who self-manage their symptoms, or women who seek care from other practitioners will not be identified as cases of problematic menopause in a primary care setting. Secondly, we were unable to distinguish local (vaginal) estrogen and systemic (oral) estrogen, some of false positive cases might be women who received estrogen therapy for other reasons, though given the age restriction and methods used to create the reference set, we are confident that most estrogens were prescribed for symptoms of genitourinary syndrome of menopause (GSM). Lastly, our case definition identified cases that have been diagnosed previously by family physicians, it was not designed to predict new cases or to support clinical decisions.

## Conclusion

We reported on the use of machine learning methods to develop a case definition for problematic menopause from primary care EMR data. The definition requires that the chart of a woman who is 45–60 years of age contains at least two instances of ICD-9 code 627 (menopausal and postmenopausal disorders) or any of its sub-codes in the billing, encounter diagnosis, or problem list within any 24 month period, or at least one medication in the ATC class G03CA (natural and semisynthetic estrogens) [35]. The validation of this case definition yielded high sensitivity of 81.5% (95% CI: 76.3-85.9%), specificity of 93.5% (91.9-94.8%), PPV of 73.8% (68.3-78.6%), and NPV of 95.7% (94.4-96.8%). The findings from this study could lead to further work on the epidemiology of problematic menopause and its symptom management in primary care settings, and ultimately aid primary care providers in the treatment of patients with problematic menopause.

## Data Availability

The data used in this analysis are available from the Canadian Primary Care Sentinel Surveillance Study (CPCSSN) upon reasonable request to SR, with permission from CPCSSN. The Menopause and Treated or Problem Menopause - Guidance Document used for chart review is available upon request to SR.

## Abbreviations

ATC: Anatomical Therapeutic Chemical
CaRT: Classification and Regression Trees
CI: Confidence Interval
COPD: Chronic Obstructive Pulmonary Disease
CPCSSN: Canadian Primary Care Sentinel Surveillance Network
EMR: Electronic Medical Record
ICD-9-CM: International Classification of Diseases– 9th Version (Clinical Modification)
kBF: *k*-Best Features
ML: Machine Learning
NCV: Nested 10-Fold Cross-Validation
NPV: Negative Predictive Value
PPV: Positive Predictive Value
RFE: Recursive Feature Elimination
