# Correction: Development and validation of a case definition for problematic menopause in primary care electronic medical records

**DOI:** 10.1186/s12911-023-02333-x

**Published:** 2023-10-19

**Authors:** Anh N. Q. Pham, Michael Cummings, Nese Yuksel, Beate Sydora, Tyler Williamson, Stephanie Garies, Russell Pilling, Cliff Lindeman, Sue Ross

**Affiliations:** 1 Department of Rehabilitation Medicine, University of Alberta, Edmonton, Canada; 2 Department of Community Health Sciences, University of Calgary, Calgary, Canada; 3 Faculty of Pharmacy and Pharmaceutical Sciences, College of Health Sciences, University of Alberta, Edmonton, Canada; 4 Department of Obstetrics and Gynecology, University of Alberta, Edmonton, Canada; 5 Department of Family Medicine, University of Calgary, Calgary, Canada; 6 Department of Family Medicine, University of Alberta, Edmonton, Canada

Correction: Pham et al. BMC Medical Informatics and Decision Making (2023) 23:202

10.1186/s12911-023-02298-x

Following the publication of the original article [1], it was brought to our attention that an error had been introduced during typesetting. In the article [1], Eq. 2 was the duplication of Eq. 1.

The correct Eq. 2 should have been:

$${F}_{1}=2{\left(\frac{1}{{S}_{n}}+\frac{1}{\text{P}\text{P}\text{V}}\right)}^{-1}$$

The original article [1] has been updated.
